# Effects of the peripheral CB_1_ receptor antagonist JD5037 in mono— and polytherapy with the AMPK activator metformin in a monocrotaline-induced rat model of pulmonary hypertension

**DOI:** 10.3389/fphar.2022.965613

**Published:** 2022-09-02

**Authors:** Patryk Remiszewski, Anna Pędzińska-Betiuk, Krzysztof Mińczuk, Eberhard Schlicker, Justyna Klimek, Janusz Dzięcioł, Barbara Malinowska

**Affiliations:** ^1^ Department of Experimental Physiology and Pathophysiology, Medical University of Bialystok, Bialystok, Poland; ^2^ Department of Pharmacology and Toxicology, University of Bonn, Bonn, Germany; ^3^ Department of Human Anatomy, Medical University of Bialystok, Bialystok, Poland

**Keywords:** AMP-activated protein kinase, cannabinoid 1 receptor, JD5037, metformin, monocrotaline, polytherapy, pulmonary arterial hypertension

## Abstract

Pulmonary hypertension (PH) is a disease leading to increased pressure in the pulmonary artery and right heart failure. The adenosine monophosphate-activated protein kinase (AMPK) activator, metformin, has a protective effect against PH. CB_1_ receptor blockade reduces the number of pathological alterations in experimental lung fibrosis. The current study evaluates the effect of the peripheral cannabinoid CB_1_ receptor antagonist JD5037 in mono- and polytherapy with metformin in rat monocrotaline-induced mild PH. Animals received metformin (100 mg/kg), JD5037 (3 mg/kg), or a combination of both once daily for 21 days. Monocrotaline (60 mg/kg) increased right ventricular (RV) systolic pressure (RVSP), led to RV and lung hypertrophy and remodeling, and decreased oxygen saturation. Metformin partially restored the monocrotaline-induced effects, i.e., decreased RVSP, increased oxygen saturation, and counteracted cardiac fibrotic, hypertrophic, and inflammatory changes. JD5037 modified parameters related to inflammation and/or fibrosis. Only polytherapy with metformin and JD5037 improved Fulton’s index and coronary artery hypertrophy and tended to be more effective than monotherapy against alterations in RVSP, oxygen saturation and coronary artery tunica media vacuolization. In conclusion, monotherapy with JD5037 does not markedly influence the PH-related changes. However, polytherapy with metformin tends to be more efficient than any of these compounds alone.

## Introduction

Pulmonary hypertension (PH) is a rare disease characterized by increased pulmonary arterial pressure over 25 mmHg at rest, which leads to right heart failure and premature death. Among five classified etiological types of PH, pulmonary arterial hypertension (PAH) is the least common but most widely studied one. Its pathophysiology is mainly based on the remodeling of the pulmonary vascular bed with high pulmonary vascular resistance (Simonneau et al., 2019; Mandras et al., 2020; Levine, 2021; Sommer et al., 2021; Zhao et al., 2021). Despite its low incidence and prevalence (ca. 5.8 and 51 per million, respectively) (Leber et al., 2021), PAH is considered a significant issue due to its high mortality (5-years survival rate from 68% in patients with low risk to 23% in the high-risk group) (Hoeper et al., 2017). Currently available pharmacological treatment options, although significantly improving survival statistics (Levine, 2021; Sommer et al., 2021), are not able to cure PAH. Therefore, new strategies that cover more than vasodilation are required (Zhang et al., 2020; Sommer et al., 2021; Zolty, 2021). Here, activation of 5′-adenosine monophosphate (AMP)-activated protein kinase (AMPK) and blockade of peripheral cannabinoid type 1 (CB_1_) receptors will be considered.

AMPK is a sensor of the cellular energy status that senses low cell adenosine triphosphate (ATP) concentration (e.g. during exercise, oxidative stress and hypoxia) (Rodríguez et al., 2021). AMPK is being considered a possible target for PAH treatment, since the endothelial AMPK is downregulated in pulmonary arteries of patients with PAH and knockout of AMPK in mice may accelerate PH progression (Omura et al., 2016). Indeed, AMPK activation has beneficial effects on PAH (Zhao et al., 2021; Zolty, 2021; Flores et al., 2022). E.g., metformin, which is not only important for type 2 diabetes mellitus therapy (Lv and Guo, 2020) but also represents the canonical AMPK activator, decreased proliferation of pulmonary artery smooth muscle cells derived from PAH patients (Dean et al., 2016) and rats exposed to endothelin-1 (Wu et al., 2014) or galectin-3 (Zhang et al., 2021). Moreover, it improved the carbachol-induced relaxation and reduced the phenylephrine-induced contraction of pulmonary arteries isolated from rats with PH elicited by hypoxia (Agard et al., 2009; Deng et al., 2020). In chronic experiments, metformin attenuated PH in rats or mice induced by monocrotaline (MCT) (Agard et al., 2009; Li et al., 2016; Zhai et al., 2018; Yoshida et al., 2020; Sun et al., 2022), hypoxia (Agard et al., 2009; Omura et al., 2016; Liu Y. et al., 2019) and sugen/hypoxia (Dean et al., 2016; Zhang et al., 2018). Moreover, the results of the first of two (Zhao et al., 2021) clinical studies evaluating metformin in PAH have shown that the compound may improve right ventricle (RV) function and reverse some negative metabolic changes in the course of PAH (Brittain et al., 2020). However, other studies suggest that AMPK might facilitate hypoxic pulmonary vasoconstriction (Evans and Hardie, 2020). Thus, further research determining the role of AMPK in PH is still needed (Deng et al., 2020; Zhao et al., 2021).

CB_1_ receptors are part of the endocannabinoid system. Their high abundance in the brain is responsible for the psychoactive effect of Δ^9^-tetrahydrocannabinol, but thanks to its distribution in almost all body tissues, CB_1_ receptors can modulate many different functions (Fowler, 2021). Peripheral overactivity of CB_1_ receptors induces cardiac, pulmonary, liver and kidney fibrosis and promotes inflammation and/or oxidative stress (Puhl, 2020; Kicman et al., 2021), and therefore their blockade could become a potential therapeutic strategy (Cinar et al., 2020). In addition, CB_1_ receptor expression is increased in the lungs from patients with idiopathic pulmonary fibrosis, which was connected with marked alveolar interstitial collagen deposition (Cinar et al., 2017), and in fibrotic lungs of patients with Hermansky-Pudlak syndrome (Cinar et al., 2021). It has been proved, that genetic deletion of CB_1_ receptors or chronic administration of their peripheral antagonists (AM6545 or JD5037) mitigates inflammation and fibrosis and increases animal survival in murine pulmonary fibrosis induced by radiation (Bronova et al., 2015) or bleomycin (Cinar et al., 2017), in experimental liver fibrosis (Tan et al., 2020) and experimental diabetic nephropathy (Barutta et al., 2018).

The gold standard for PAH treatment nowadays is an early combined therapy with compounds that affect different pharmacological targets (Klinger et al., 2019; Mandras et al., 2021; Mayeux et al., 2021; Sommer et al., 2021; Tettey et al., 2021). This kind of therapeutic procedure is superior to therapies with the agents alone as suggested by several meta-analyses based on clinical studies (Sommer et al., 2021) and is recommended by expert guidelines (Klinger et al., 2019). Moreover, PAH is a multi-factorial disease, and the vasoconstriction of pulmonary arteries as the main target of current treatments for PAH appears insufficient. Searching for new targets and treatment strategies not involving pulmonary vasodilation, we studied the influence of separate and combined administration of the peripheral CB_1_ receptor antagonist JD5037 and the AMPK activator metformin on the MCT-induced PH in rats.

## Materials and methods

### Animals

All procedures and experimental protocols were performed in accordance with the European Directive (2010/63/EU) and with the approval of the local Animal Ethics Committee in Olsztyn (Poland) (approval codes 74/2020, 9/WNP/WDO/2021 and 39/WNP/2021). Rats were obtained from the Centre for Experimental Medicine of the Medical University of Bialystok (Poland). They had free access to chow and water and were kept under a 12:12 h light-dark cycle and constant temperature (21 ± 2°C) and humidity (55 ± 5%).

### Protocol and experimental groups

On day 0, male Wistar rats were given a single subcutaneous (s.c.) injection of monocrotaline (MCT) (60 mg/kg in a volume of 3 ml/kg) to induce pulmonary hypertension (PH) (Sadowska et al., 2020). The studied compounds were administered in a preventive regimen. Controls (CTR) received a s.c. injection of an equal volume of 0.9% NaCl. From day 1 onward, metformin (100 mg/kg; MET), JD5037 (3 mg/kg; JD), the combination of metformin and JD5037 or their vehicles (veh; *metformin*: 0.9% NaCl, 5 ml/kg; *JD5037*: DMSO, Tween 80 and 0.9% NaCl 4:1:95, 5 ml/kg) were administered to CTR and PH rats by oral gavage every 24 h for 21 days.

There were 8 groups of animals: 1) CTR + veh, 2) CTR + MET, 3) CTR + JD, 4) CTR + MET + JD, 5) PH + veh, 6) PH + MET, 7) PH + JD and 8) PH + MET + JD. Originally, there were even 10 groups. However, the groups “CTR + veh for MET” and “CTR + veh for JD” and the groups “PH + veh for MET” and “PH + veh for JD” were combined since the respective values did not differ significantly (Table 1).

**TABLE 1:** Influence of metformin (MET), JD5037 (JD) and their combination (MET + JD) on physiological parameters of monocrotaline-induced pulmonary hypertensive (PH) rats and their normotensive controls (CTR).

Group Parameter		CTR + veh	CTR + MET	CTR + JD	CTR + MET + JD	PH + veh	PH + MET	PH + JD	PH + MET + JD
	*n*	18–20	9–10	8–10	9–10	17–20	9–10	8–10	8–10
Body weight (g)	day 0	313 ± 3	313 ± 6	315 ± 4	316 ± 4	313 ± 2	313 ± 6	316 ± 6	315 ± 4
	day 22	365 ± 5^$$$^	363 ± 8^$$$^	357 ± 6^$$$^	354 ± 6^$$$^	347 ± 4^$$$,^ *	342 ± 8^$$$^	345 ± 6^$$$^	347 ± 4^$$$^
SBP (mmHg)	day 1	130 ± 3	122 ± 4	122 ± 4	132 ± 6	133 ± 4	137 ± 5	135 ± 10	137 ± 6
	day 22	131 ± 4	131 ± 7	132 ± 9	134 ± 6	131 ± 4	125 ± 2	125 ± 7	129 ± 6
HR	by pulse oximeter	286 ± 2	267 ± 6	283 ± 5	316 ± 9**	293 ± 5	288 ± 10	295 ± 7	296 ± 11
(beats/min)	by catheter	267 ± 6	255 ± 3	249 ± 5	275 ± 6	264 ± 5	261 ± 6	259 ± 4	253 ± 7
dP/dt_max_ (mmHg/s)		1487 ± 45	1482 ± 44	1588 ± 59	1477 ± 51	1804 ± 46***	1730 ± 91	1703 ± 53	1645 ± 68
dP/dt_min_ (mmHg/s)		-1054 ± 30	-1076 ± 54	-1089 ± 55	-1080 ± 35	-1400 ± 56***	-1240 ± 99	-1270 ± 61	-1180 ± 72
Rectal temperature (°C)		35.7 ± 0.2	35.7 ± 0.2	35.6 ± 0.2	36.4 ± 0.3	36.1 ± 0.2	36.2 ± 0.3	35.7 ± 0.3	35.9 ± 0.2
Heart weight/BW (mg/g)		2.83 ± 0.04	2.80 ± 0.04	2.79 ± 0.08	2.79 ± 0.05	2.89 ± 0.06	2.87 ± 0.07	2.91 ± 0.06	2.86 ± 0.07
RA weight/BW (mg/g)		0.106 ± 0.004	0.103 ± 0.004	0.100 ± 0.006	0.118 ± 0.011	0.115 ± 0.005	0.109 ± 0.009	0.117 ± 0.008	0.110 ± 0.010
LA weight/BW (mg/g)		0.069 ± 0.003	0.079 ± 0.006	0.081 ± 0.003	0.078 ± 0.003	0.069 ± 0.003	0.072 ± 0.003	0.070 ± 0.002	0.074 ± 0.003
RV weight/BW (mg/g)		0.448 ± 0.008	0.444 ± 0.014	0.457 ± 0.013	0.462 ± 0.016	0.491 ± 0.012	0.498 ± 0.022	0.510 ± 0.013	0.460 ± 0.016
LV + S weight/BW (mg/g)		1.71 ± 0.02	1.67 ± 0.02	1.70 ± 0.04	1.71 ± 0.03	1.68 ± 0.03	1.73 ± 0.02	1.69 ± 0.04	1.70 ± 0.03
Kidney weight/BW (mg/g)		3.51 ± 0.07	3.55 ± 0.05	3.52 ± 0.06	3.52 ± 0.07	3.59 ± 0.07	3.68 ± 0.06	3.69 ± 0.08	3.61 ± 0.07
Blood glucose (mg/dl)		128 ± 2	125 ± 2	129 ± 4	141 ± 6*	125 ± 3	128 ± 4	132 ± 4	137 ± 7

MET (100 mg/kg), JD5037 (3 mg/kg) or their combination were administered by oral gavage once daily for 21 days (controls received vehicles instead). Measurement of SBP, in conscious and of HR, dP/dt_max/min_ and rectal temperature in anaesthetized animals. Parameters were determined 24 h after the last injection, i.e., on day 22; body weight and SBP, were also determined on days 0 and 1, respectively. Data are mean ± SEM. *p < 0.05, **p < 0.01, ***p < 0.001 significantly different from CTR + veh, ^$$$^ *p* < 0.001 significantly different from day 0.

*n*, the number of rats per group; in CTR + veh and PH + veh, *n* was double as high because both groups, which did not differ in their results, were combined.

veh–vehicle; SBP, systolic blood pressure; HR, heart rate; dP/dt_max_, dP/dt_min_–rate of rise/decrease of right ventricular pressure; BW, body weight; RA, right atrium; LA, left atrium; RV, right ventricle; LV + S–left ventricle + septum.

Animals were randomly allocated to the experimental groups and did not differ with respect to weight (see Table 1) and age (9–11 weeks old) at the beginning of the protocol.

### Determination of cardiovascular parameters in conscious rats

Systolic blood pressure (SBP) was measured using the non-invasive tail-cuff method with the Non-Invasive Blood Pressure Controller (ADInstruments, Sydney, Australia) after the administration of MCT or its vehicle before the first dose of metformin and/or JD5037 or their vehicle and after completion of the study (24 h after the last injection).

### Determination of blood glucose level

Blood glucose level was measured in blood samples from the lateral tail vein using the Accu-Chek blood glucose meter (Roche Diabetes Care GmbH, Mannheim, Germany).

### Determination of right ventricular systolic pressure

After induction of anaesthesia with ketamine and xylazine (i.p., 1 ml of ketamine 100 mg/ml + 100 μl of xylazine 20 mg/ml; 300 μl per 250 g of body weight)*,* a pressure catheter with a sensor for the measurement of the right ventricular systolic pressure (RVSP), HR and the rate of rise/decrease of right ventricular pressure (dP/dt_min/max_) (SPR-320 Mikro-Tip, Millar, Houston, TX, USA) was pushed forward through the right jugular vein and placed in the right ventricle. Data were acquired using LabChart 7.3.7 Pro (ADInstruments, Sydney, Australia).

### Determination of blood oxygen saturation

Blood oxygen saturation and heart rate (HR) were measured using a pulse oximeter (MouseSTAT^®^ Jr Rodent Pulse Oximeter and Heart Rate Monitor with Rat Paw Pulse Oximeter Sensor, Kent Scientific Corporation, Torrington, CT, USA) attached to the left front paw of the animal right after anaesthesia and after placing the rat on a heating pad (Bio-Sys-Tech, Białystok, Poland).

### Determination of rectal temperature

Rectal temperature was measured using a rectal probe transducer (RDT 100; Bio-Sys-Tech, Białystok, Poland) right after anaesthesia.

### Determination of organ weight and hypertrophy indexes

After RVSP determination, the heart and lungs were removed. Next, the right ventricle (RV), left ventricle with septum (LV + S), right (RA) and left (LA) atria, left lung and left kidney were separated and weighed. Right ventricle hypertrophy was expressed in two ways: as Fulton’s index, which is RV weight to LV + S weight (RV/LV + S) and as right ventricular hypertrophy index, which is RV weight to body weight of the animal. Lung hypertrophy index was expressed as left lung weight to body weight of the animal.

### Histopathology

After separation and weighing, RV and right lung were fixed with 10% buffered formalin. The tissue was paraffin-embedded and cross-sectioned at 5-µm thickness; sections were subjected to hematoxylin and eosin (H&E) staining.

For the quantification of the pulmonary artery vascular wall thickness, the % wall area was calculated from the area of smooth muscle (total area of the vessels - lumen area of the vessels), divided by the total area of vessels (Jiang et al., 2021)**.** Ten vessels per lung were counted. Only arteries with a diameter ranging from 50 to 150 µm were included in the statistical calculations. Mean vessel size was comparable among groups and amounted to approximately 74 µm.

Histopathological evaluations of hearts and lungs were performed by a veterinary pathologist with a specialization in the pathology of laboratory animals. The criteria for histopathological evaluation were based on the International Harmonization of Nomenclature and Diagnostic Criteria for Lesions in Rats and Mice (INHAND) guidelines (Berridge et al., 2016) developed by ESTP, STP, BSTP and JSTP. Histopathological assessments were made in a system describing the organ, histological structure, pathological change, and the severity of the pathological change on a scale of 0–4.

### Western blot analysis

After separation and weighing, the left lung was rinsed with 0.9% saline, drained and snap-frozen with liquid nitrogen and stored at −80°C. After pulverization, samples were homogenized in a protein extraction reagent containing a cocktail of protease inhibitors and centrifuged at 10,000 × g for 10 min at 4°C. In addition, protein concentration was measured using the bicinchoninic acid method (BCA) with bovine serum albumin (BSA) as a standard. Subsequently, homogenates were reconstituted in Laemmli buffer with a reducing agent. The same amounts of protein (30 µg) were loaded on a polyacrylamide gel. After electrophoresis, proteins were transferred onto a nitrocellulose membrane. Next, the membranes were blocked to minimalize non-specific signal and incubated overnight at 4°C with the corresponding primary antibodies in appropriate dilutions: galectin-3 (1:5,000) and TGF-β1 (1:1000). Thereafter, membranes were incubated with the appropriate secondary antibody conjugated to horseradish peroxidase. After adding a suitable substrate, protein bands were detected using a ChemiDoc™ XRS+ System (Bio-Rad, Hercules, CA, United States). Then, Western blots were quantified densitometrically with ImageJ 1.53p software (National Institutes of Health, Bethesda, MD, USA). The expression of selected target proteins was standardized to β-actin expression.

### Statistical analysis

Results are expressed as mean values ± standard error of the mean (SEM) or medians with an interquartile range. At the beginning, each of the 10 groups consisted of 10 rats. However, the final n was 6–20 because 1) the groups receiving the solvent for MET and JD5037 were combined (no significant differences between the values), 2) measurement of hemodynamic parameters failed in a few cases and/or 3) outliers (values deviating from the mean by more than plus/minus three standard deviations) were excluded. To obtain an accurate group size (*n* = 5) in WB analysis and meet the requirement of running all samples on a single gel, normotensive groups receiving the treatment were excluded (no changes observed in preliminary experiments). All data were subjected to the Kolmogorov-Smirnov test to assess the distribution of values. If the data were normally distributed, the (parametric) one-way analysis of variance (ANOVA) with Bonferroni’s multiple comparison test for multiple groups or paired Student’s t-test for comparison within the group was carried out. Data subjected to ANOVA were followed by Bonferroni’s post hoc tests only when the F value attained *p* < 0.05 and there was no significant inhomogeneity of variances. Histopathological scoring was performed on the basis of an ordinal scale and for this reason the nonparametric Kruskal-Wallis test with Dunn’s post hoc test was used (Gibson-Corley et al., 2013). Statistical analysis was performed using Graph Pad Prism 5 (GraphPad Software, La Jolla, CA, United States).

### Drugs

JD5037 (2S)-2-[[[(4S)-5-(4-chlorophenyl)-4-phenyl-3,4-dihydropyrazol-2-yl]-[(4-chlorophenyl)sulfonylamino]methylidene]amino]-3-methylbutanamide (530481) from MedKoo Biosciences, Morrisville, NC, United States; metformin (PA-03–2747-P-25G) from POL-AURA, Różnowo, Poland; monocrotaline (C2401) from Sigma-Aldrich, Burlington, MA, USA; ketamine (5909997022796) from Biowet, Puławy, Poland; xylazine (5909997021911) from Vetoquinol Biowet, Gorzów Wielkopolski, Poland; anti-galectin-3 antibody (ab76245), anti-TGF-β1 antibody (ab179695) and anti-β-actin antibody (ab8227) from Abcam, Cambridge, United Kingdom.

## Results

### General

As shown in Table 1, the body weight of rats was similar among all groups on day 0 and increased during the subsequent 22 days. No mortality following MCT administration was observed. On day 22, the body weight in the control group (CTR; no MCT, no drugs) was higher by about 5% than in the corresponding PH group. Systolic blood pressure (SBP) measured non-invasively before the first and after the last dose of the studied compounds was comparable in normotensive and PH groups. Although PH did not affect heart rate (HR), combined administration of JD5037 (3 mg/kg) and metformin (100 mg/kg) significantly increased (+ 10%; measured by a pulse oximeter) or tended to increase (catheter) this parameter in the control group. The rates of rise (dP/dt_max_) and of decrease (dP/dt_min_) of right ventricular pressure increased (+21%) and became significantly more negative (−33%), respectively, in the PH vs. the control group. There was no influence of PH or therapy on rectal temperature and kidney hypertrophy index. With respect to cardiac hypertrophy indexes, PH only tended to increase the parameter of the right atrium and ventricle. The combined therapy of metformin and JD5037 tended to decrease the right ventricle hypertrophy index and to normalize the PH-induced changes in dP/dt_max_ and dP/dt_min_. Polytherapy increased blood glucose level in controls (+10%) and tended to do so in animals with PH.

### Influence of PH and drug therapies on RVSP, Fulton’s index and blood oxygen saturation

**FIGURE 1:**
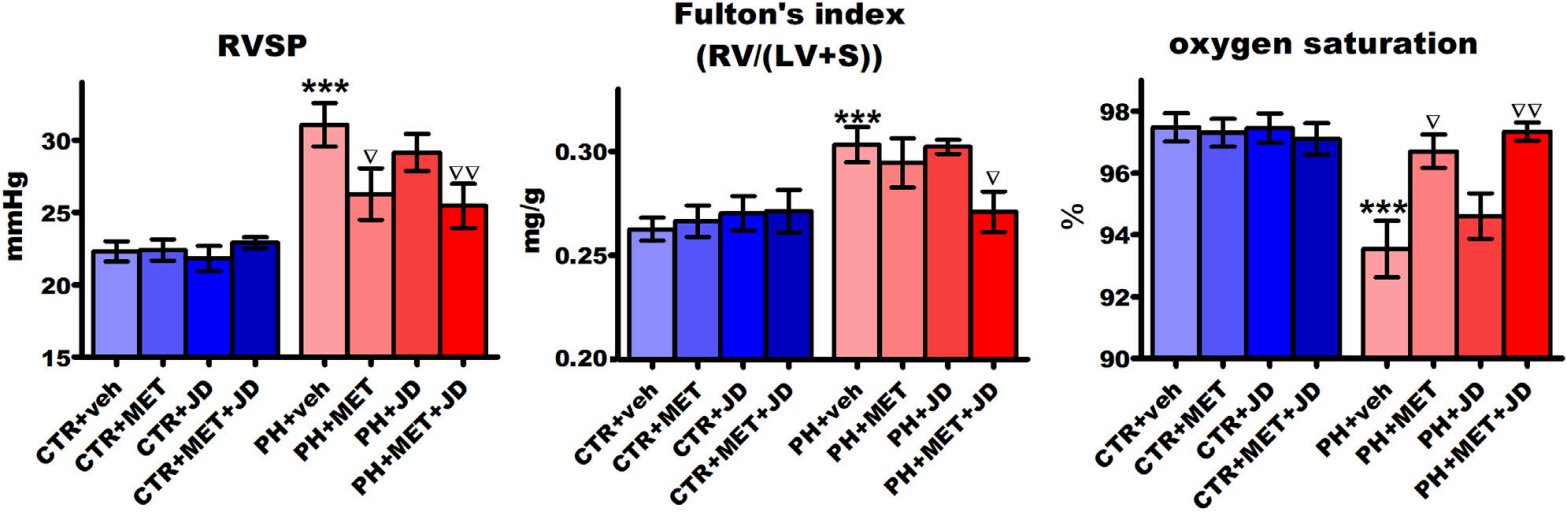
Influence of pulmonary hypertension, metformin (MET), JD5037 (JD) and/or their combination on right ventricular systolic pressure (RVSP), Fulton’s index (RV/(LV + S)) and oxygen saturation in monocrotaline-induced pulmonary hypertensive (PH) rats and their normotensive controls (CTR). MET (100 mg/kg), JD5037 (3 mg/kg) or their combination were administered by oral gavage once daily for 21 days; controls received vehicles instead. Data are expressed as mean ± SEM; *n* = 9–20; ***p < 0.001 significantly different from CTR + veh; ^∇^ *p* < 0.05; ^∇∇^ *p* < 0.01 significantly different from PH + veh. RV–right ventricle; LV + S–left ventricle + interventricular septum.

As shown in Figure 1, right ventricular systolic pressure (RVSP) and Fulton’s index were higher (by 39% and 16%, respectively) and blood oxygen saturation was lower (by 4%) in the PH than in the normotensive control group. Chronic 21-day administration of metformin partially normalized the changes associated with PH, i.e. decreased RVSP by 15% and increased oxygen saturation by 3%. There was no effect of metformin on Fulton’s index or of JD5037 on any of the three parameters in the PH group. The combined therapy of metformin and JD5037 tended to have stronger effects on the PH-induced changes than the monotherapies, i.e. decreased RVSP by 18% and increased oxygen saturation by 4%. A statistically significant effect on Fulton’s index (decrease by 11%) was observed after combination therapy only.

### Influence of PH and drug therapies on the right ventricle

**FIGURE 2:**
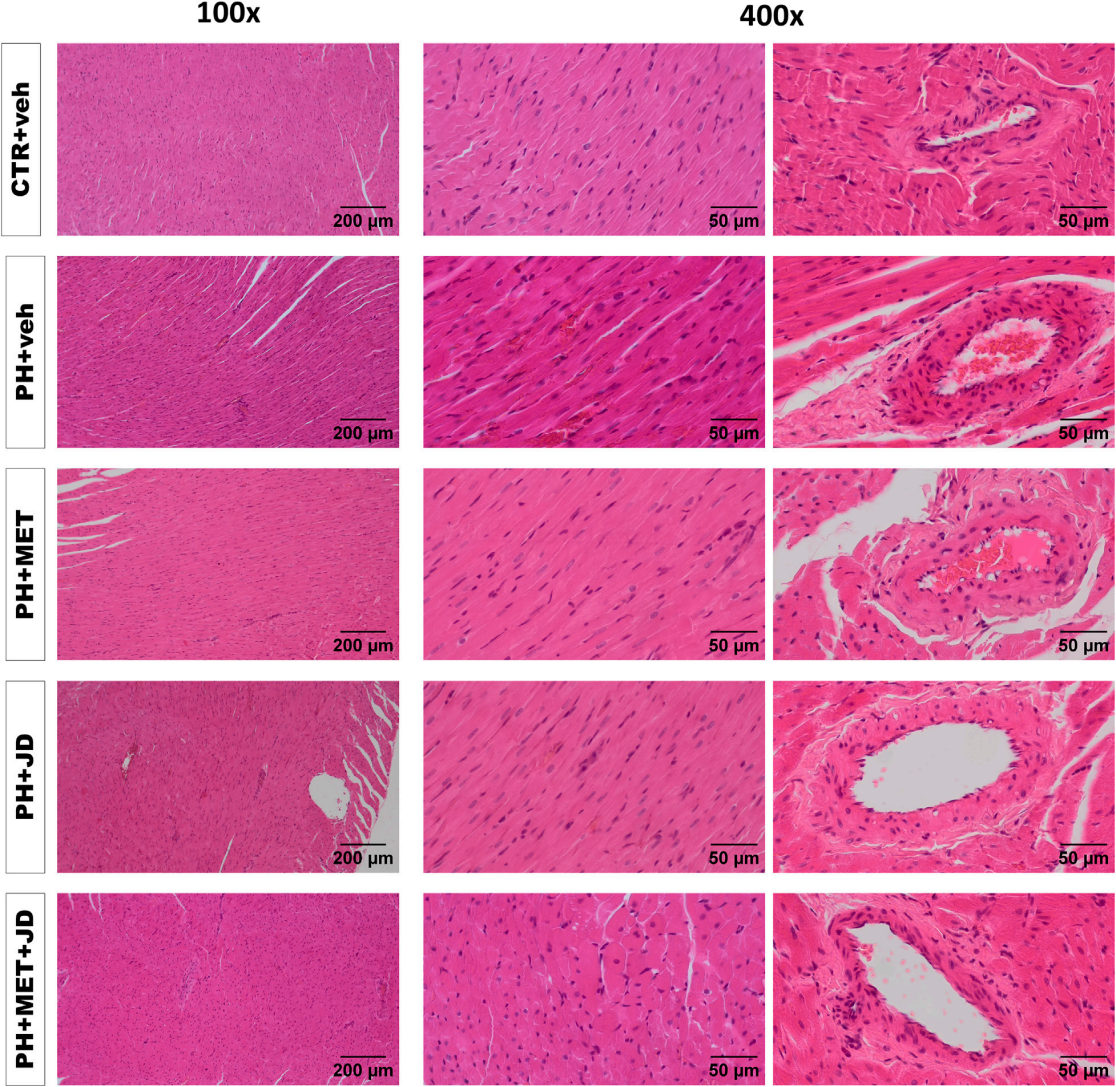
Influence of pulmonary hypertension, metformin (MET), JD5037 (JD) and/or their combination on right ventricular remodeling in monocrotaline-induced pulmonary hypertensive (PH) rats and their normotensive controls (CTR). Representative hematoxylin and eosin-stained right ventricle images (100x and 400x magnification); quantitative evaluation is provided in Table 2. MET (100 mg/kg), JD5037 (3 mg/kg) or their combination were administered by oral gavage once daily for 21 days; controls received vehicles (veh) instead.

As shown in Table 2 and Figure 2, PH caused changes in right ventricle tissue assessed by histological scoring. Compared to the control group, significant alterations can be observed in cardiomyocytes (hypertrophy and hypereosinophilia of cytoplasm, i.e., an increase in the intensity of eosin staining), myocardium (increased waviness of muscle fibers, hyperplasia of connective tissue and extravasation) and coronary arteries (hypertrophy and vacuolization of tunica media and increased infiltration of mononuclear cells). Tendencies of changes connected with PH vs. control can be found for almost every histopathological parameter assessed. In addition, only tendencies in Masson trichrome collagen staining were observed (Supplementary Table S1). Metformin or JD5037 administered alone decreased hyperplasia of connective tissue in myocardium, vacuolization of tunica media of coronary arteries and infiltration of plasmacytic and/or mast cells in pericardium, which were or tended to be elevated in PH. JD5037, unlike metformin, decreased mast cell infiltration in myocardium and the infiltration of mononuclear cells in myocardium and coronary arteries. Combined therapy with metformin and JD5037 influenced the same parameters as in monotherapies with similar or better results, except for tunica media hypertrophy of coronary arteries, for which it was the only effective treatment.

**TABLE 2:** Influence of metformin (MET), JD5037 (JD) and their combination (MET + JD) on histopathological right ventricular scoring of monocrotaline-induced pulmonary hypertensive (PH) rats and their normotensive controls (CTR).

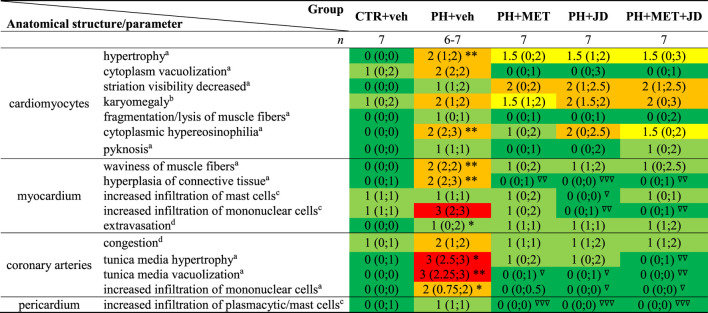

MET (100 mg/kg), JD5037 (3 mg/kg) or their combination were administered by oral gavage once daily for 21 days; controls received vehicles (veh) instead. Data are based on 6-7 rats per group and are expressed as median of scores ranging from 0 to 4 with an interquartile range. *p < 0.05, **p < 0.01 significantly different from CTR + veh. ^∇^ *p* < 0.05, ^∇∇^ *p* < 0.01, ^∇∇∇^ *p* < 0.001 significantly different from PH + veh. The colors correspond to the median values of the scoring of the group: dark green (0), light green (1), yellow (1.5), orange (2) and red (3).

a Scoring scale type B5: 0—no pathological changes; 1—minimal disruptions in architecture/structure, ×40 objective; 2—moderate disruptions in architecture/structure, ×40 objective; 3—minimal/mild disruptions in architecture/structure, ×10 objective; 4—moderate/marked disruptions in architecture/structure, ×10 objective.

b Scoring scale type A5: 0—0–5%; 1—6–25%; 2–26–50%; 3–51–75%; 4–76–100%.

c Scoring scale type C3: 0—no pathological changes, ×40 objective; 1—up to 2 foci; 2—3-4 foci; 3—5-6 foci; 4—>6 foci.

d Scoring scale type B4: 0—no pathological changes; 1—minimal/mild increase of changes, ×40 objective; 2—mild severity of changes, ×10 objective; 3—moderate severity of changes, ×10 objective; 4—moderate/marked severity of changes, ×4 objective.

### Influence of PH and drug therapies on the lungs

**FIGURE 3:**
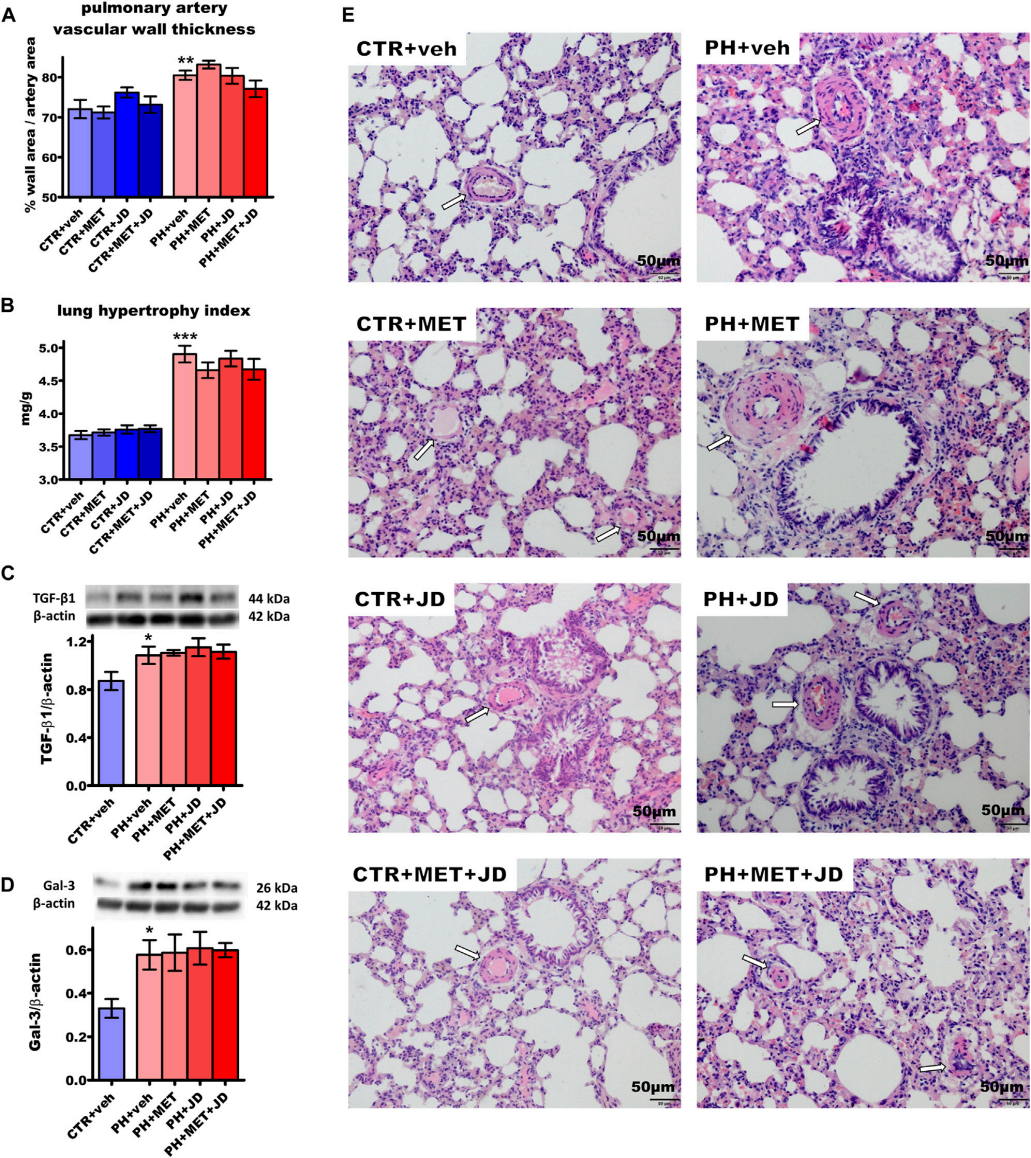
Influence of pulmonary hypertension, metformin (MET), JD5037 (JD) and/or their combination on pulmonary remodeling in monocrotaline-induced pulmonary hypertensive (PH) rats and their normotensive controls (CTR). **(A)** pulmonary artery vascular wall thickness, **(B)** lung hypertrophy index (expressed as lung weight to body weight) and expression of **(C)** transforming growth factor β1 (TGF-β1) and **(D)** galectin-3 (Gal-3) determined by Western blot (WB) technique, **(E)** representative hematoxylin and eosin-stained lung images (200x magnification). β-actin served as a loading control in WB. Arrows on images show the location of the vessels. MET (100 mg/kg), JD5037 (3 mg/kg) or their combination were administered by oral gavage once daily for 21 days; controls received vehicles (veh) instead. Data are expressed as mean ± SEM; *n* = 9–20 **(A–B)**, *n* = 5 **(C,D)**; *p < 0.05; **p < 0.01; ***p < 0.001 significantly different from CTR + veh.

As shown in Figures 3A–D, PH increased pulmonary artery vascular wall thickness by 12%, the lung hypertrophy index by 33%, transforming growth factor β1 (TGF-β1) expression in the lung by 25% and galectin-3 expression by 75% compared to the normotensive control. In addition, PH rats showed an increased medial wall thickness, stenosis of arteries, inflammatory cells infiltration and thickening of interalveolar partitions (for representative images, see Figure 3E). However, the degree of muscularization in pulmonary vessels was not increased by PH (Supplementary Table S1). PH-induced alterations were not influenced by metformin, JD5037 or their combination in a statistically significant manner; only tendencies were observed. Thus, metformin tended to diminish the lung hypertrophy index and the combined therapy, in addition, tended to reduce pulmonary artery vascular wall thickness.

## Discussion

This study shows for the first time that the peripheral CB_1_ receptor antagonist JD5037 has beneficial effects in a monocrotaline protocol of the rat associated with mild pulmonary hypertension. Moreover, the beneficial effects of another protective agent, the AMPK activator metformin, are further increased or only become significant in combination with JD5037.

### Methodological considerations

Using MCT, we applied one of the most accepted preclinical rodent models of established PH (also used for the development of PAH-targeted therapies). Rats were preferred since rapid metabolism of MCT occurs in mice (Dignam et al., 2022). A dose of 60 mg/kg is sufficiently high to lead to the pathological features of PH (Bonnet et al., 2017; Dignam et al., 2022; Jama et al., 2022). Metformin was administered at 100 mg/kg for 21 days since this protocol had a beneficial effect on PH induced by hypoxia (i.p., 21 days) (Liu et al., 2019) and sugen/hypoxia (orally, 21 days) (Dean et al., 2016) in rats. JD5037 was applied at 3 mg/kg for 21 days since similar protocols proved effective against liver fibrosis (orally, 2 or 8 weeks) (Tan et al., 2020) or obesity-induced chronic kidney disease (orally, 28 days) (Udi et al., 2020) in rodents.

### Induction of pulmonary hypertension

Our study shows that PH is developing during 21 days after MCT injection and leads to changes of cardiac and pulmonary parameters. RVSP, the main determinant of PH development, was elevated by about 40%. The value of the pressure in the right ventricle of about 30 mmHg is not high and allows to classify the resulting hypertension as mild or early-stage PH. Very similar observations have been made in experiments conducted on rats of comparable age from exactly the same source as ours by another group (Hołda et al., 2020a; Hołda et al., 2020b). In addition, analogous values of RVSP were observed previously in rats using the RV catheterization technique and even in some longer-lasting protocols the effect was not further increased (Agard et al., 2009; Dai et al., 2010; Ou et al., 2010; Gubrij et al., 2014; Meghwani et al., 2018; Jin et al., 2019; Cao et al., 2020; Videja et al., 2021; Sun et al., 2022). However, we would like to underline that the pathophysiological basis of early PAH is not sufficiently understood. Very rapid progression of the disease that leads to RV failure induced by MCT (to a lesser extent also by other factors that cause experimental PH) may prevent the development of compensatory mechanisms that normally occur in humans and is being considered as one of the weaknesses of this model. Many effective therapies in animal studies have not been translated into clinical trials, because they do not completely reflect human PAH (Dignam et al., 2022). Moreover, the recently proposed lowering of the diagnostic threshold for pulmonary hypertension brings a new challenge, namely the search for treatments that would be effective in patients with lower pulmonary pressure values. All presently used therapies have been studied in cases with advanced PH and it is unclear if currently available treatment schedules will be helpful for them (Hoeper and Humbert, 2019; Stewart et al., 2020; Sommer et al., 2021). That is why research into PH in its early stages is extremely important to find the right treatment options for a new group of patients.

In our previous study (Sadowska et al., 2020) we had used 5–8 week-old rats but we preferred 9–11 week-old animals in the present one to avoid premature deaths. Kawade et al. (2021) compared 7 and 20-week-old rats and found that MCT led to a much higher survival rate but also induced less severe PH in older compared to younger rats. This observation also translated into our experience and that of another group using animals from exactly the same source as ours (Hołda et al., 2020a); in both instances, no animal mortality was observed because of the development of mild PAH.

The increase in RVSP was associated with a significant enlargement of RV, expressed as Fulton’s index, and a tendency of an increase in RV weight/body weight ratio and heart weight/body weight ratio, like in our previous paper (Sadowska et al., 2020). The PH animals also showed increased rates of rise (dP/dt_max_) and decrease (dP/dt_min_) of right ventricular pressure. These data suggest that in our model RV maintains its function by increasing contractile (inotropic) and lusitropic action and is still in a compensatory phase (Vélez-Rendón et al., 2018; Oknińska et al., 2021). In addition, the histopathological results support functional and organ hypertrophic changes in RV. Thus, PH led to cardiomyocyte hypertrophy, cardiomyocyte hypereosinophilia and a wavy arrangement of myocardial fibers, fibrotic and inflammatory modifications (also of the coronary arteries) and tissue damage.

PH also caused pulmonary alterations, such as lung hypertrophy and an increase of pulmonary arterial wall thickness, which had been previously described, among others, also by our group (Sadowska et al., 2020). In the present paper, the PH-induced increase in pulmonary arterial wall thickness cannot be related to a proliferation of vascular smooth muscle cells since PH did not lead to changes in muscularization. The macroscopic findings were reflected by biochemical alterations in lung tissue, i.e. an increase in TGF-β1 and Gal-3 expression. Gal-3 in PH is associated with an impairment of redox balance and induction of inflammation, both of which contribute to vascular fibrosis and remodeling (Fulton et al., 2019; Barman et al., 2021). In addition, Gal-3 interacts with many signaling molecules, including TGF-β1, which induces fibrosis during chronic inflammatory diseases (Weiskirchen et al., 2019), plays a crucial role in the PAH pathogenesis and is a promising target to treat this disease (Sanada et al., 2021; Andre et al., 2022). Both parameters are therefore early predictors of tissue remodeling that have been observed in our model.

To summarize, hemodynamic, histological and biochemical parameters determined in our study 3 weeks after MCT administration are characteristic for mild PH only. The most severe changes (including evident fibrosis) are observed mainly 4 weeks after MCT administration (Xu et al., 2018; Hołda et al., 2020a; Padrez et al., 2022).

### Effects of metformin and/or JD5037 on PH

In previous studies (Agard et al., 2009; Li et al., 2016; Zhai et al., 2018; Sun et al., 2019; Yoshida et al., 2020), metformin was proved effective in attenuating the MCT-induced PH-related changes in rats, such as increased RVSP, Fulton’s index, pulmonary arterial thickness or lung tissue collagen deposition. In our hands, metformin partially restored the PH-induced RVSP increase and the decreased blood oxygen saturation. Moreover, in the histological part of our study, it attenuated the fibrotic, hypertrophic and inflammatory alterations induced by PH. The lack of a significant influence on macroscopic parameters related to RV and pulmonary vascular hypertrophy may result from the (too high) age of the animals at the time of PH induction and/or the (too short) duration of the experimental protocol, since metformin was applied for 21 days in our hands as opposed to 28–30 days in the publications listed above. The fact that metformin lowers RVSP (in fact, partially prevents it from increasing), but does not affect Fulton’s index may come as a surprise. However, it should be noted that RV hypertrophy is not necessarily solely due to an increased afterload. Another reason may be the direct toxic effect of MCT on the heart including right ventricle hypertrophy and myocarditis (Jasińska-Stroschein, 2021; Dignam et al., 2022). Metformin has been used for years as an antidiabetic agent (Lv and Guo, 2020) and is a safe and well-tolerated drug, as also suggested by our experiments on control and MCT-treated rats. Its lack of an effect on blood glucose levels suggests that its beneficial effects on some of the PH-related alterations are not associated with its anti-diabetic properties.

The CB_1_ receptor antagonist JD5037 is a quite new compound and has been tested so far in animal models only, in which, like in our own experiments, no serious adverse effects were found (Kale et al., 2019). The fact that the activity of JD5037 is restricted to the periphery is advantageous since CB_1_ receptor antagonists penetrating the blood-brain barrier may lead to severe central side effects; for this reason, rimonabant had even to be withdrawn from the market (Cinar et al., 2020). In our hands, chronic administration of JD5037 alone improved or tended to improve some microscopic and biochemical parameters related to inflammation (infiltration of immune cells in myocardium, coronary arteries and pericardium) and/or fibrosis (hyperplasia of myocardial connective tissue and tunica media hypertrophy). These data are not surprising, since CB_1_ receptor overstimulation or overactivity leads to cardiac dysfunction, inflammation or oxidative stress (Puhl, 2020) and pulmonary injury with inflammation and fibrosis (Zawatsky et al., 2020; Cinar et al., 2022). In addition, CB_1_ receptor blockade was effective as an anti-inflammatory and anti-fibrotic strategy in animal models (Bronova et al., 2015; Cinar et al., 2017; Barutta et al., 2018; Tan et al., 2020). On the other hand, one has to admit that in our PH model JD5037 did not act against several aspects of the MCT-induced PH and, in particular, did not modify the main changes in RVSP, Fulton’s index or oxygen saturation.

Combined 21-day therapy with metformin and JD5037 was found to be an effective strategy against the PH-related alterations in RVSP, Fulton’s index, oxygen saturation or the histopathological hypertrophic and inflammatory changes in RV tissue. Only the dual treatment decreased Fulton’s index and the hypertrophy in the media of coronary arteries and tended to decrease RV/body weight ratio. In addition, the PH-induced increase in RVSP and the decrease of blood oxygen levels tended to be further improved after polytherapy when compared to monotherapy with metformin. Moreover, the combination therapy tended to decrease pulmonary artery vascular thickness and to normalize dP/dt. An interesting observation is that both mono- and polytherapy do not modify biochemical predictors of lung tissue remodeling (TGFβ-1 and Gal-3). In general, all pulmonary parameters are not or only weakly amenable to therapy.

To summarize, mechanistically, both metformin and JD5037 were effective against hallmarks of mild PH connected with inflammation and remodeling. Furthermore, in both cases myocardial cellular and/or vascular antihypertrophic activity was found. The reason for the superior effect of the combination therapy against most parameters of MCT-induced PH may be that two complementary mechanisms act in parallel. However, a potential additive effect of both compounds is conceivable as well since JD5037 (and other CB_1_ receptor inhibitors) may act also via activation of AMPK (Liu et al., 2019). On the other hand, two explanations can be excluded. Thus, the combination therapy increased rather than decreased both blood glucose level and HR although changes were very slight. When given alone, neither the CB_1_ receptor antagonist AM251 (Weresa et al., 2019) nor metformin (Zilov et al., 2019) modifies cardiovascular parameters. Metformin is well-known for its glycemia-reducing activity in diabetic individuals (Lv and Guo, 2020) and JD5037 improves glucose metabolism in obese (Tam et al., 2012; Cinar et al., 2014; Knani et al., 2016; Liu et al., 2019) and diabetic (Hinden et al., 2018) mice. In addition, the CB_1_ receptor antagonists rimonabant (Christopoulou and Kiortsis, 2011) and taranabant (Kipnes et al., 2010) used in clinical trials of type 2 diabetes reduced the level of glycated hemoglobin in metformin-treated patients. Probably an unknown interaction is responsible for the unexpected increase in glucose and HR and further research is needed to elucidate its mechanism.

### Limitations of the study

In the present study, we examined the potential preventive effects of a three-week-long administration of metformin (100 mg/kg), JD5037 (3 mg/kg) or their combination on MCT-induced PH in male rats. Thus, one should keep in mind that other results may be obtained if 1) experimental PH is induced by other stimuli, e.g., sugen plus hypoxia; 2) a therapeutic rather than a preventive paradigm is used; 3) compounds are administered at a higher dose or for a longer time; 4) the end-stage PH is studied (obtained by a longer period of PH development, the use of younger rats and/or another strain, e.g., Sprague-Dawley) and 5) the PH is induced in female animals. Despite the fact that PAH develops predominantly in women (Docherty et al., 2018; Hester et al., 2019), we used male rats since the development of the MCT-induced PH is less pronounced in female animals (Frump et al., 2021).

## Conclusion

Chronic 21-day combined administration of metformin (100 mg/kg) and JD5037 (3 mg/kg) attenuated most of the mild PH-induced cardiopulmonary alterations and tended to be more efficient than any of the monotherapies alone. Quite recently third-generation CB_1_ receptor antagonists have been synthesized, i.e., compounds combining peripherally restricted CB_1_ receptor antagonism with an additional target. AMPK activation is suggested as a possible secondary target for such hybrid molecules (Cinar et al., 2020) and dual compounds combining peripheral CB_1_ antagonism and AMPK activation have been presented in an abstract form very recently (Iyer et al., 2022). Our results argue in favour of further studies dedicated to such hybrid compounds. In such studies, the significance of the combined therapy of peripheral CB_1_ receptor antagonism plus AMPK activation in pulmonary arterial hypertension, both in its early and end-stage phases, should be taken into consideration. Moreover, one should keep in mind that the third week after the monocrotaline administration is critical for PH development and might result in the induction of both early and end-stage PH.

## Data Availability

The original contributions presented in the study are included in the article/Supplementary Material, further inquiries can be directed to the corresponding author.
