# Supplementary material for: Effects of the peripheral CB1 receptor antagonist JD5037 in mono— and polytherapy with the AMPK activator metformin in a monocrotaline-induced rat model of pulmonary hypertension
Source: Front Pharmacol. 2022 Sep 2;13:965613. doi: 10.3389/fphar.2022.965613 (PMC9479636; doi:10.3389/fphar.2022.965613)
Supplement: Supplementary file 1 [file DataSheet1.PDF]

Supplementary Material

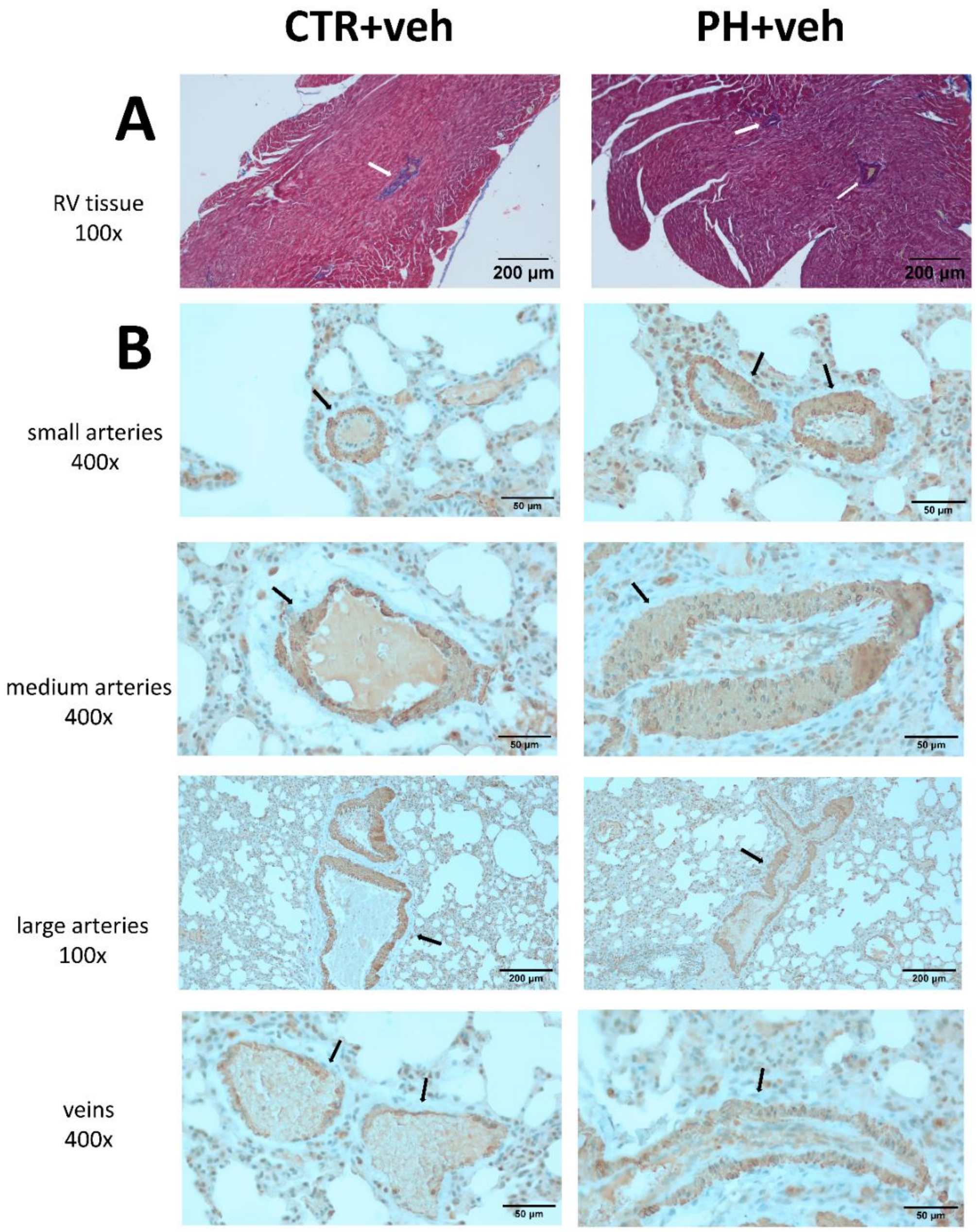

Supplementary Figure 1. Representative Masson trichrome-stained right ventricle (RV) images (**A**) and alpha smooth muscle actin immunohistochemistry-stained lung images of different types of vessels (**B**) of control (CTR+veh) and pulmonary hypertensive (PH+veh) animals (100x or 400x magnification, as indicated); quantitative evaluation is provided in Supplementary Table 1. Arrows indicate blood vessels.

Supplementary Table 1. Comparison of control (CTR+veh) and monocrotaline-induced pulmonary hypertensive (PH+veh) groups in lung and right ventricle (RV) histopathological and/or biochemical parameters. Only tendencies were observed.

Parameter	CTR+veh	*n*	PH+veh	*n*
**Masson trichrome staining (%) in right ventricle**				
- between bunches of cardiomyocytes	2.3 ± 0.3	7	2.5 ± 0.2	7
- around the coronary vessels	5.3 ± 1.0	7	8.8 ± 3.3	7
**α smooth muscle actin staining scoring (0-4) in lungs**				
- small arteries	2 (2;2)	7	2 (2;2)	7
- medium arteries	2 (2;3)	7	2 (2;3)	7
- large arteries	3 (2;3)	7	3 (3;3)	6
- veins	2 (2;3)	7	2 (2;2)	7
**Collagen I Western blot (arbitrary units) in lungs**	0.85 ± 0.09	5	1.0 ± 0.09	5

Monocrotaline (60 mg/kg) was injected s.c. at day 0. Tissues were isolated from animals on day 22. Data are presented as mean ± SEM of the percent area of the positive color reaction in Masson's trichromatic staining in the right ventricle, median with an interquartile range of scoring points in α smooth muscle actin staining in lungs and mean ± SEM of arbitrary units of collagen I/β-actin ratio in lungs. Scoring scale: absent expression (**0**); weak expression (**1**); moderate expression (**2**); high expression (**3**); very high expression (**4**).

1. Supplementary materials and methods

1.1 Histopathology

1.1.1 Alpha smooth muscle actin staining

Lungs were fixed with 10% buffered formalin. The tissue was paraffin-embedded and cross-sectioned at 5-µm thickness; sections were subjected to alpha smooth muscle actin (αSMA) immunohistochemical staining. Immunohistochemistry (IHC) grading based on intensity and frequency of staining results was performed by two independent investigators without knowledge of the clinicopathological features of the animals. The staining intensity was scored as negative (0), weak (+1), moderate (+2), or strong (+3). The frequency of positive cells in specific areas was scored as negative (0), less than 25% (+1), 25–50% (+2), 51–75% (+3), or more than 75% (+4). IHC grades were calculated by multiplying the intensity score by the frequency score as follows: −, absent expression (**0**); +, weak expression (**1**); ++, moderate expression (**2**); +++, high expression (**3**); or ++++, very high

expression (**4**). Vessels were assigned to the appropriate group according to their size. Large arteries – vessels of the lung cavity and their direct branches, visualized at x100 magnification or in the entire field of view at x400 magnification; medium arteries – vessels imaged at x400 magnification with an outer diameter of more than 1/3 of the field of view; lumen to wall thickness ratio greater than 1:1 in favor of lumen; small arteries – vessels imaged at x400 magnification with the morphology of thick-walled resistance vessels; outer diameter of the vessel less than 1/3 of the field of view; a lumen to wall thickness ratio of less than 1:1 in favor of the vessel wall; veins – vessels with a typical vein morphology, mainly imaged under x400 magnification.

1.1.2 Masson trichrome staining

As part of the histological technique, sections of right ventricle tissue were stained with Masson's trichromatic staining for collagen of connective tissue, using the kit Masson Trichrome with aniline blue (Bio-Optica, Milan, Italy; cat. no. 40211). The interstitial collagen volume fraction (CVF) in the myocardium (defined as the area of the positive color reaction in Masson's trichromatic staining (pink-purple) surface) was quantified in two localizations: 1) between bunches of cardiomyocytes and 2) around the coronary vessels to the total area of myocardium. The parameter is given as a percentage of positive color reaction staining in the entire surface of the myocardium. The described measurements were performed on histological photos of myocardium taken in a light microscope Olympus BX41 (Tokyo, Japan) with an Olympus DP12 camera magnification of 100 (x10 at the lens and x10 at the eyepiece). Morphometric measurements were made in the Zen 3.0 program (Carl Zeiss, Oberkochen, Germany) (Blue edition).

1.1 Western blot

The same procedure of the Western blot technique was used as in the main text of the article, except for not using a reducing agent in the sample buffer. The anti-collagen I antibody (Santa Cruz Biotechnology, Dallas, TX, USA; cat. no. sc-293182) was used in a dilution of 1:750.

Western blot original images

Sample order:

- standard (row 1)
- CTR+veh (rows 2, 7, 12, 17, 22)
- PH+veh (rows 3, 8, 13, 18, 23)
- PH+MET (rows 4, 9, 14, 19, 24)
- PH+JD (rows 5, 10, 15, 20, 25)
- PH+JD+MET (rows 6, 11, 16, 21, 26)

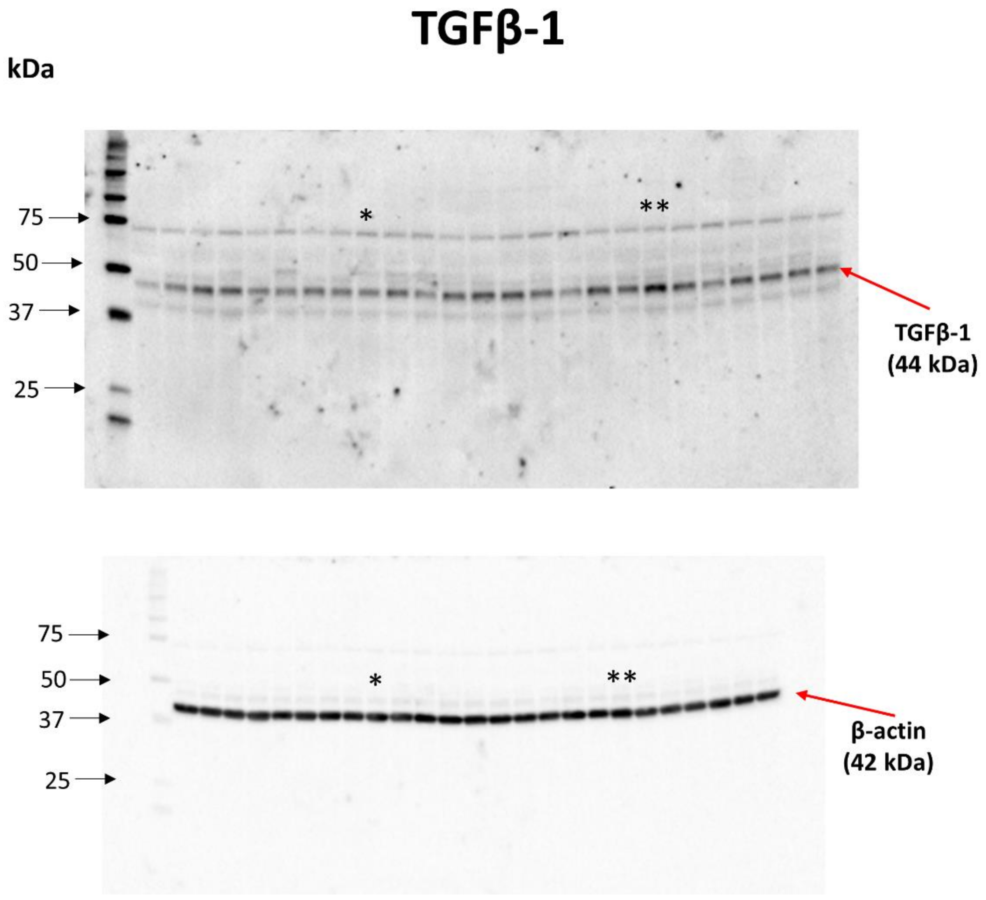

Supplementary Figure 2. Original images of Western blot analysis of transforming growth factor β-1 (TGFβ-1) and β-actin (loading control). The 10th and 20th rows are marked by * and **, respectively.

Western blot original images

Sample order:
- standard (row 1)
- CTR+veh (rows 2, 7, 12, 17, 22)
- PH+veh (rows 3, 8, 13, 18, 23)
- PH+MET (rows 4, 9, 14, 19, 24)
- PH+JD (rows 5, 10, 15, 20, 25)
- PH+JD+MET (rows 6, 11, 16, 21, 26)

Gal-3

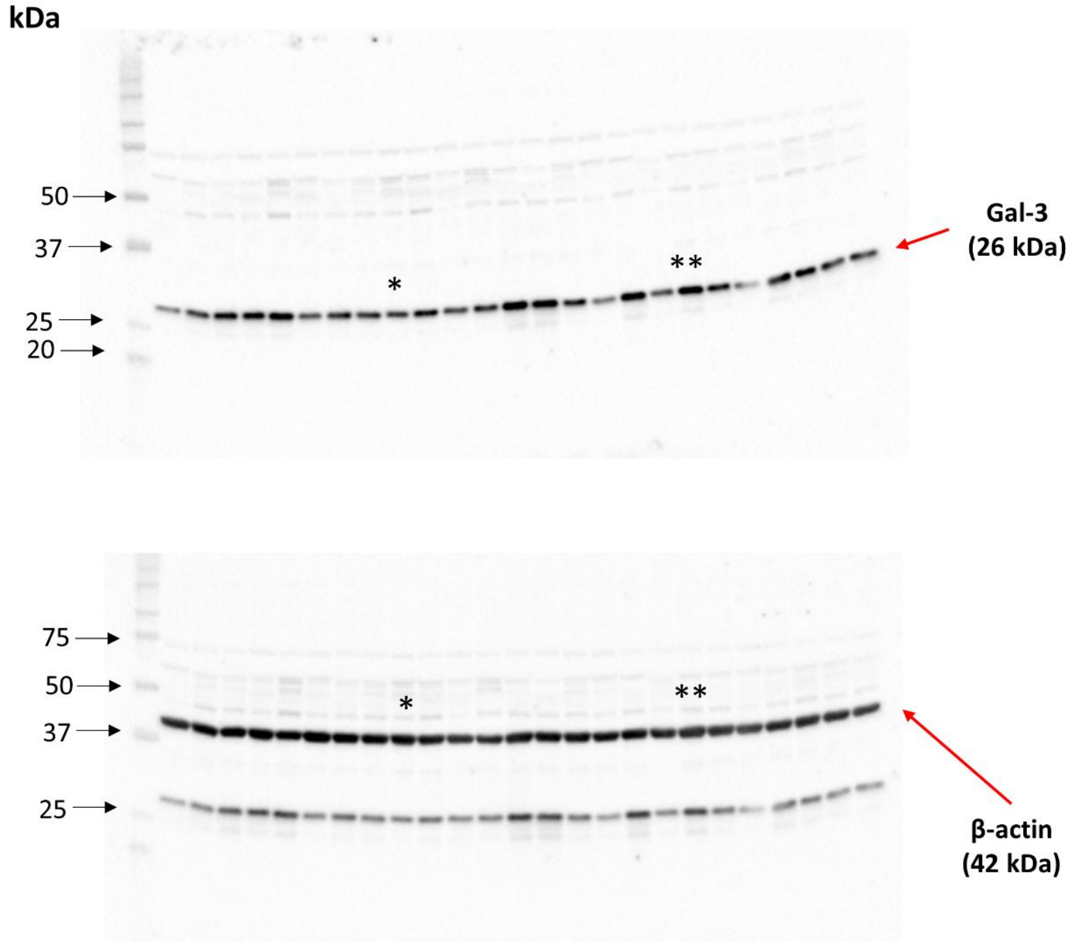

Supplementary Figure 3. Original images of Western blot analysis of galectin-3 (Gal-3) and β-actin (loading control). The 10th and 20th rows are marked by * and **, respectively.
